# Cost-effectiveness analysis comparing companion diagnostic tests for EGFR, ALK, and ROS1 versus next-generation sequencing (NGS) in advanced adenocarcinoma lung cancer patients

**DOI:** 10.1186/s12885-020-07240-2

**Published:** 2020-09-14

**Authors:** Luciene Schluckebier, Rosangela Caetano, Osvaldo Ulises Garay, Giuliana T. Montenegro, Marcelo Custodio, Veronica Aran, Carlos Gil Ferreira

**Affiliations:** 1 Fundação do Câncer, 212 - Centro, Rio de janeiro, 20231-048 Brazil; 2 Universidade do Estado do Rio de Janeiro (UERJ), Rio de Janeiro, Brazil; 3 Instituto de Efectividad Clinica y Sanitaria (IECS-CONICET), Buenos Aires, Argentina; 4 Roche Diagnóstica, Buenos Aires, Argentina; 5 AstraZeneca Medical Division Brasil, Cotia, São Paulo, Brazil; 6 Instituto Estadual do Cérebro Paulo Niemeyer, R. do Rezende, 156 - Centro, Rio de Janeiro, 20231-092 Brazil; 7 Oncoclínicas, Rio de Janeiro, Brazil

**Keywords:** Lung cancer, NGS, NSCLC, Diagnostic methods, Health economics, EGFR, ALK, ROS1

## Abstract

**Background:**

The treatment of choice for advanced non–small cell lung cancer is selected according to the presence of specific alterations. Patients should undergo molecular testing for relevant modifications and the mutational status of EGFR and translocation of ALK and ROS1 are commonly tested to offer the best intervention. In addition, the tests costs should also be taken in consideration. Therefore, this work was performed in order to evaluate the cost-effectiveness of a unique exam using NGS (next generation sequencing) versus other routinely used tests which involve RT-PCR and FISH.

**Methods:**

The target population was NSCLC, adenocarcinoma, and candidates to first-line therapy. Two strategies were undertaken, strategy 1 corresponded to sequential tests with EGFR RT-PCR, then FISH for ALK and ROS1. Strategy 2 differed from 1 in that ALK and ROS1 translocation testing were performed simultaneously by FISH. Strategy 3 considered single test next-generation sequencing, a platform that includes EGFR, ALK and ROS1 genes. A decision tree analysis was used to model genetic testing options. From the test results, a microsimulation model was nested to estimate survival outcomes and costs of therapeutic options.

**Results:**

The use of NGS added 24% extra true cases as well as extra costs attributed to the molecular testing. The ICER comparing NGS with sequential tests was US$ 3479.11/correct case detected. The NGS improved a slight gain in life years and QALYs.

**Conclusion:**

Our results indicated that, although precise, the molecular diagnosis by NGS of patients with advanced stage NSCLC adenocarcinoma histology was not cost-effective in terms of quality-adjusted life years from the perspective of the Brazilian supplementary health system.

## Background

Advanced lung cancer has played a key role in the development of medicines aimed for individualized therapy. Currently, it is recommended that all patients who are candidates for chemotherapy treatment should undergo molecular testing to determine the best treatment clinically available [1–3].

The Epidermal Growth Factor Receptor (EGFR) was the first antigenic target used as a guideline for targeted lung cancer therapy, followed by other targeting markers, such as KRAS (*Kirsten rat sarcoma viral oncogene homolog*) and fusion of EML4-ALK1 (*echinoderm microtubule-associated protein-like 4 – anaplastic lymphoma kinase*), along with other ongoing clinical trials, such as MET, BRAF, RET (*Echinoderm Microtubule Associated Protein like 4-AL-Kinase 1*) and ROS1 (*receptor tyrosine kinase* 1) [4]. These genes are frequently mutated in non-small cell lung cancer (NSCLC) with variable frequencies**:** EGFR [5, 6], ALK [7]; ROS1 [8, 9] and RET [10]. The majority of these mutations are mutually exclusive, and sensitive to targeted therapies available at the clinic level.

Different mutations can be identified through different genotyping methods that cover “screening” or “targeting” [11]. Also, methods may vary depending on the type of material available for examination, coverage of mutations, performance, accuracy, technical complexity and costs [12]. Making a poor choice of test can, aside from wasting tissue samples, compromise the entire treatment. This might occur since less accurate tests might lead to inadequate results, ineffective therapy, and lost time and resources.

Technologies such as sequencing, PCR, in situ hybridization (FISH) and immunohistochemistry (IHC), among others, were developed and are being used for the clinical evaluation of oncogenic markers. Due to technical limitations and the small amount of material obtained from biopsies, none of these techniques can be scaled to meet the increasing number and variety of genomic changes. This has led to the development of parallel multi-genic DNA sequencing platforms such as next-generation sequencing (NGS), that allows for the simultaneous analysis of hundreds of genetic alterations in a single test [13].

The major impediment for effective implementation of individualized therapy is the access to companion tests and drugs, due to the high costs that health systems generally cannot afford. Brazil is a middle-income country that had an estimated 208 million inhabitants and where 31,270 new lung cancer cases were expected in 2018 [14]. Within the country, two health subsystems coexist, one of public and universal financing and the private health insurance sector covering around 47 m people (about 25% of the population) [15]. The regulation defines the compulsory coverage of private health care plans throughout the national territory for oral anti-neoplastic treatments, ensuring access to treatment with gefitinib, erlotinib, afatinib and crizotinib [16]. The Law also comprises companion tests without defining which method should be used. The 3rd generation EGFR inhibitors (e.g. osimertinib) and the 2nd and 3rd generation ALK inhibitors (e.g. alectinib, ceritinib, brigatinib, lorlatinib) were not included in this model, neither as options for first-line therapy nor at the time of progression on first-line inhibitors, since these options were not registred in Brazil at the time, and they did not have any payment or reimbursement support in our country.

The NGS technique has been raising expectations about the possibility of performing a single test to define prognosis and treatment. However, this technique is associated with high costs, raising doubts about whether its dissemination is cost-effectiveness for health systems. Few countries evaluated the varying strategies for the use of molecular tests [17, 18]. To this end, this work compared, in terms of cost and effectiveness, the inclusion of varying companion diagnostic tests in a single test-treatment model. The tests included recommended techniques currently used in clinical practice for identifying mutations in EGFR (e.g. RT-PCR), and ALK and ROS1 (e.g. FISH), to the single test using NGS for tumor tissue samples. All analyses were done from the perspective of the Brazilian private health insurance sector.

## Methods

### Study population and treatment strategies

A cost-effectiveness study was done in reference to the population of adult patients affected with adenocarcinoma of NSCLC stage IV. The economic analysis considered the long-term effects of technical performance and the accuracy of the following molecular testing strategies used for tumor tissue biopsies:

Strategy 1: RT-PCR for mutation identification of the EGFR gene. If negative, the individual is sent for ALK gene fusion testing; should this test also be negative, the patient continues on to ROS1 gene testing. Strategy 2: differs from strategy 1 in that ALK and ROS1 translocation testing are performed simultaneously by FISH. Strategy 3: considers new intervention along with next-generation sequencing, with a platform that includes EGFR, ALK and ROS1 genes in a single test.

**Fig. 1:**
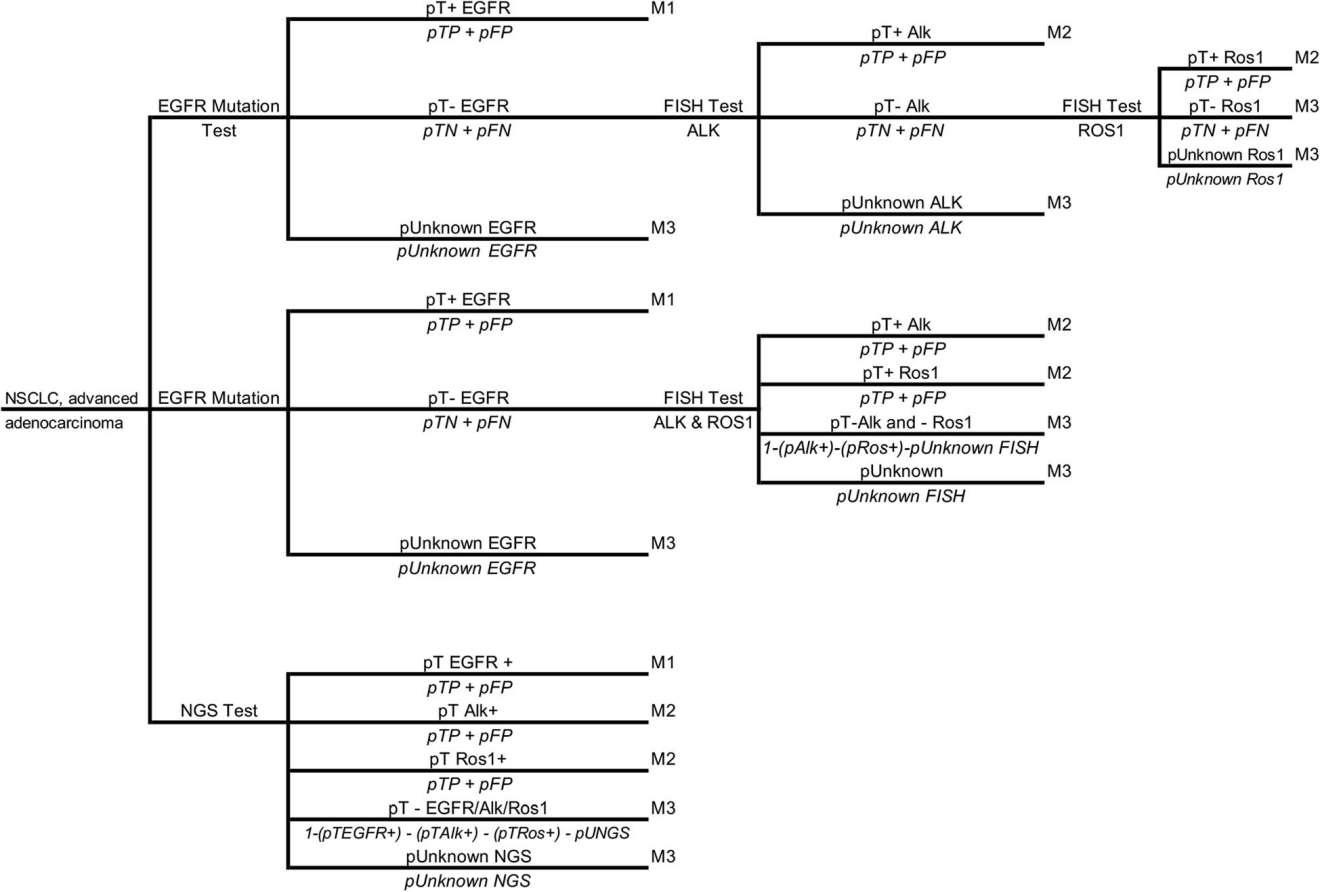
Decision tree model comparing companion diagnostics in sequence versus NGS. Legend: NSCLC: non-small cell lung cancer; pT+: proportion of positive tests (TP + FP); pT-: proportion of negative results (TN + FN); pTP: true positive probability; pFP: false positive probability; pTN: true negative probability; pFN: false negative probability. Note: pTP = prevalence x sensitivity x (1-unknow); pFP = (1-prevalence) x (1-specificity) x (1-unknow); pTN = (1-prevalence) x specificity x (1-unknow); pFN = prevalence x (1-sensitivity) x (1-unknow)

A decision tree model was built comparing the three strategies based on prevalence of genetic alterations, accuracy, and tests performance (Fig. 1).

The transition state models were constructed based on different available information. Positive results for EGFR led to the use of gefitinib in the first line [19, 20] followed by conventional chemotherapy with pemetrexed plus cisplatin after the first progression [20, 21] and docetaxel after the second [22]. Positive diagnosis for ALK or ROS1 translocation results in the use of crizotinib in the first line [23]. After progression, the treatment regimens were as described previously. The possibility of repeating the tissue biopsy was not considered for inconclusive cases. If the tests were negative or inconclusive, treatment began with pemetrexed plus platinum in the first line [24, 25], docetaxel following progression and gemcitabine in the third line [25]. Because of the specificity of the isolated test for EGFR, individuals with false-positive EGFR results entered the simulation using TKI therapy, but with survival attributed to those with negative EGFR profile [26].

Relevant outcomes estimated in the study were the costs associated with molecular diagnosis and treatment, correctly diagnosed cases (true positive and true negative) from the different diagnostic strategies, years of life gained and quality-adjusted life year (QALY). The time horizon is 5 years and all costs and health outcomes were discounted at an annually rate of 5% according to Brazilian health economic evaluation guidelines [27].

The parameters utilized in the model, as well as the value limits used in the sensitivity analysis, are described in Table 1. For the parameters of accuracy of the tests, literature comparing Cobas® EGFR Mutation Test (Roche), Therascreen EGFR PCR kit (Qiagen) and NGS with traditional direct sequencing from Sanger were used. The costs associated with the tests were based on the prices in the Brazilian market, and the benchmark value for payment from the Brazilian private sector (CBHPM). Treatment values were calculated using standard chemotherapy and target therapy protocols, with costs of medicines obtained from the price list released by the National Health Surveillance Agency (CMED/ANVISA) and corresponding to the month of March/2017. Additional costs associated with pre-chemotherapy (average US$ 250.00 per cycle) and room rates (US$ 33.00 per cycle) were also considered in parenteral drugs. All costs were calculated in local currency and converted to US dollars using the purchasing power parity conversion factor. The values of utilities were estimated based on responses and toxicities associated with treatment [37, 38].

**Table 1:** Summary of parameters, range and parameter distribution used in sensitivity analysis

Parameters	Reference case	Minimal value	Maximal value	Distribution	References
**Genetic alterations**					
Prevalence EGFR	0.28	0.22	0.34	Beta	[28, 29]
Prevalence Alk	0.05	0.02	0.07	Beta	[7, 28]
Prevalence Ros1	0.02	0.01	0.03	Beta	[8, 30]
**Test accuracy**					
Sensitivity EGFR	0.98	0.95	0.99	Beta	[12, 31–33]
Specificity EGFR	0.89	0.69	1.00	Beta	[12, 31–33]
Sensitivity Alk	1.00	0.75	1.00	Beta	[34]
Specificity Alk	1.00	1.00	1.00	Beta	[34]
Sensitivity Ros1	1.00	0.75	1.00	Beta	Assumption, as reference test
Specificity Ros1	1.00	1.00	1.00	Beta	Assumption, as reference test
Sensitivity NGS	0.99	0.96	1.00	Beta	[35, 36]
Specificity NGS	1.00	0.82	1.00	Beta	[35, 36]
**Test performance**					
pUnknow EGFR	0.13	0.06	0.34	Beta	[12, 31–33]
pUnknow FISH (ALK e ROS)	0.10	0.02	0.38	Beta	[37, 38]
pUnknow NGS	0.04	0.00	0.09	Beta	[39–41]
**Test costs**					
cEGFR	428.14	363.57	477.39	Gama	AMB, CBHPM - 2016; ANS,D-TISS; search from current Brazilian market price
cFISH Alk	573.70	423.11	753.77	Gama	
cFISH Ros1	564.99	423.11	753.77	Gama	
cNGS	1874.37	1502.51	2110.55	Gama	
**Drug price**					
Cisplatin 50 mg	113.57	63.11	141.95	Gama	Anvisa, Câmara de Regulação do Mercado de Medicamentos - CMED
Crizotinib 250 mg	15,084.79	11,245.02	19,298.94	Gama	
Docetaxel 80 mg	1271.57	1463.21	1589.47	Gama	
Gefitinib 250 mg	2256.31	1631.86	2820.38	Gama	
Gencitabin 1000 mg	1881.43	799.77	2351.78	Gama	
Pemetrexed 500 mg	3418.88	2484.86	4273.60	Gama	
**Utilities**					
1st line with Gefitinib (M1)	0.65	0.60	0.71	Beta	[37, 38]
1st line with Crizotinib (M2)	0.66	0.60	0.71	Beta	[37, 38]
Pemetrexed + cisplatin (M3, 1st Progression in M1 & M2)	0.62	0.56	0.67	Beta	[37, 38]
2nd or 3rd line with standard chemotherapy (2nd Progression in M1, M2, M3 and 1st Progression in M3)	0.57	0.51	0.64	Beta	[37, 38]
**Others**					
Discount rate	0.05	0.00	0.10	Beta	[27]

The incremental cost-effectiveness ratio (ICER) was calculated using the ratio of the differences between costs and QALYs among strategies. The ICER represents the incremental cost of a strategy for gaining a single unit of health benefit. To assess the degree of uncertainty of the results, the probabilistic sensitivity analysis was performed by varying all parameters within the uncertainty interval, according to their distribution (Table 1).

## Results

### Decision tree model comparing three molecular testing strategies

A decision tree model was constructed comparing the three strategies (described in the methods section) based on prevalence of genetic alterations, accuracy, and performance of the tests (Fig. 1). Our analysis show that the NGS strategy was not cost-effective compared to the others, however it displayed a higher probability of correct diagnoses (sum of true positive and negative cases) with 96.3% when compared to 72.6% for strategy 2 and 68% for strategy 1. The decision analysis model also showed that, hypothetically, performing 1000 NGS tests would yield 270 true EGFR positive cases, 50 positive cases for ALK and 15 positive cases for ROS1. On the other hand, 1000 tests using strategy 2 for EGFR mutation plus 500 FISH tests, would yield 240 true positive EGFR cases, 25 positive cases for ALK and 8 positive cases for ROS1. Regarding performance status, NGS resulted in 34 inconclusive tests, and strategy 2 with EGFR mutation kit resulted in 130 inconclusive cases, plus 55 inconclusive cases for FISH.

### State transition model of treatment options according to molecular test results

From the tree model results, a microsimulation model was designed to estimate the effectiveness of the diagnostics up through treatment, based on free survival data, progression, and overall survival rates of the different therapies. The study modelled the complete course of treatment over the entire life expectancy of the patients through a microsimulation model, which included second and third treatment lines. The clinical practices regarding the treatment lines used were selected from the therapeutic guidelines and validated based on consultation with specialists working in the Brazilian private health insurance sector to reflect the practice in the country.

**Fig. 2:**
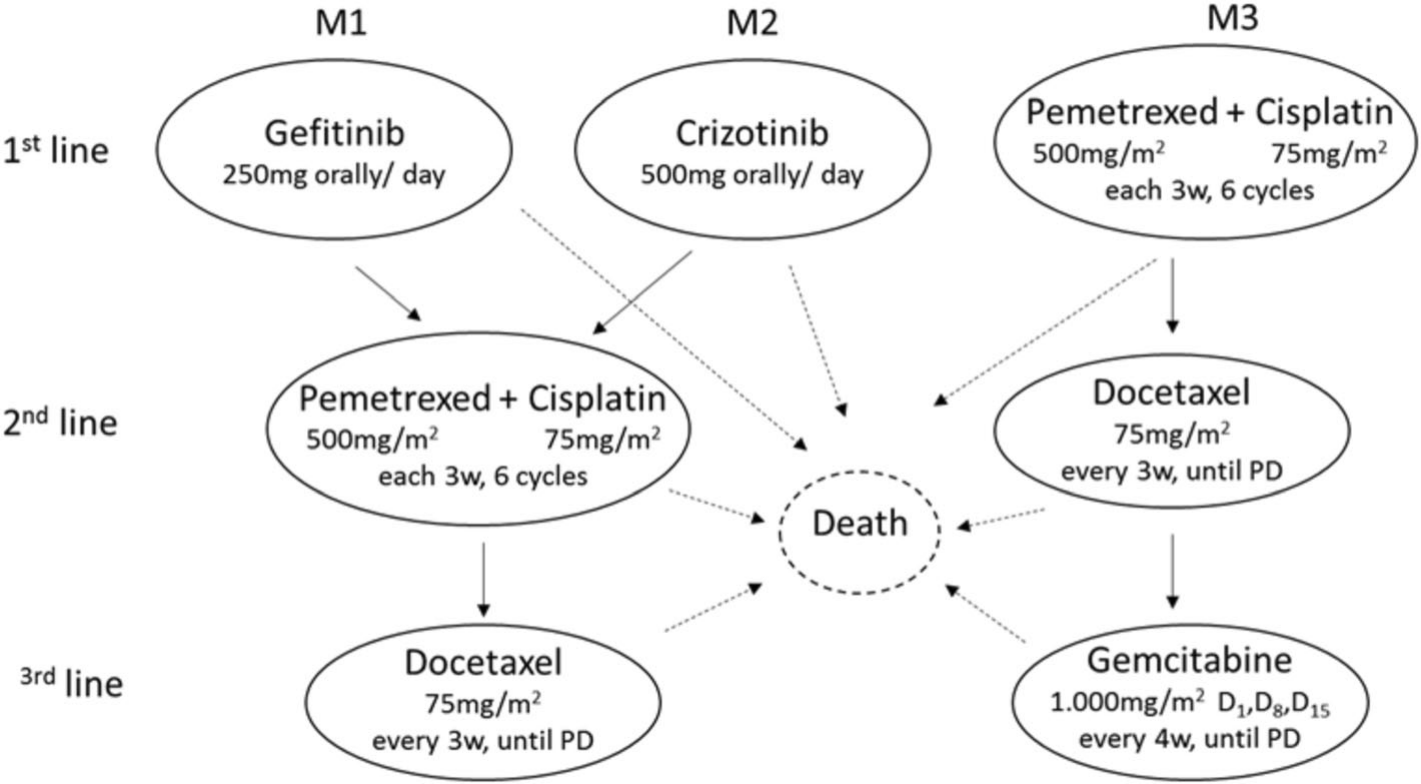
State transition model of therapeutic options according to molecular test results. Notes: These transition models were linked to each decision tree branch. Each arrow indicates the possible transitions for each state. Legend: PD: progression disease; w: week

Three transition state models were constructed, corresponding to disease progression and treatment until the third line (Fig. 2).

### Analysis of incremental cost, effectiveness in terms of correct case detected and the incremental cost-effectiveness ratio

The use of NGS identified an additional benefit of 24% of correctly diagnosed cases at an incremental cost of $800.76. The ICER comparing NGS with sequential tests was US$ 3479.11 for each correct case detected. The comparison of strategies 2 and 1 (2:1), indicated that the ICER was US$ 961.46 for each correct case detected (Table 2).

**Table 2:** Incremental cost, effectiveness in terms of correct case detected and the incremental cost-effectiveness ratio

Strategies Compared	Incremental cost	Incremental effectiveness				Sum of incremental effectiveness	ICER (cost/ true cases detected)
		TP EGFR	TP ALK	TP ROS1	TN		
1 vs 0	$ 1006.91	23.9%	2.4%	0.7%	41%	0.68	$ 1480.75
2 vs 1	$ 44.87	0.0%	0.03%	0.1%	4.5%	0.05	$ 961.46
3 vs 2	$ 822.59	3.1%	2.3%	0.7%	17.5%	0.24	$ 3479.11

*TP* true positive, *TN* true negative, *ICER* Incremental Cost-Effectiveness Ratio. Note: 0 is equal to do no tests at all (not recommended)

### The impact of the choice of diagnostic strategy on survival

**Fig. 3:**
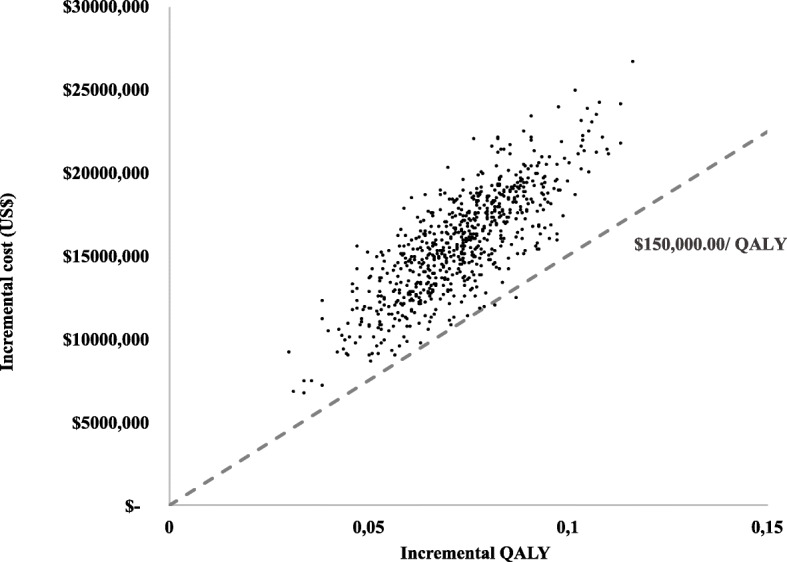
Cost-effectiveness plane plotting incremental QALY and costs comparing NGS (strategy 3) vs sequential tests (strategy 2)

The first part of the decision analysis model considered only the intermediate effects, which are the test results. To identify if, apart from the test results, the choice of diagnostic strategy has an impact on survival, a transition state model was used to consider the effectiveness of treatment. Regarding survival, the difference in incremental years of life gained, and QALYs between the strategies was very small. The cost-effectiveness plan in Fig. 3 indicates, at each point, the results of the microsimulation in terms of the incremental effectiveness and incremental cost. The calculated values for the incremental cost-effectiveness ratio from the simulations are around US $ 214,000.00 per QALY gained (IC_95%_: US$ 166,566.38 – 279,245.48).

### NGS compared to standard strategies

**Fig. 4:**
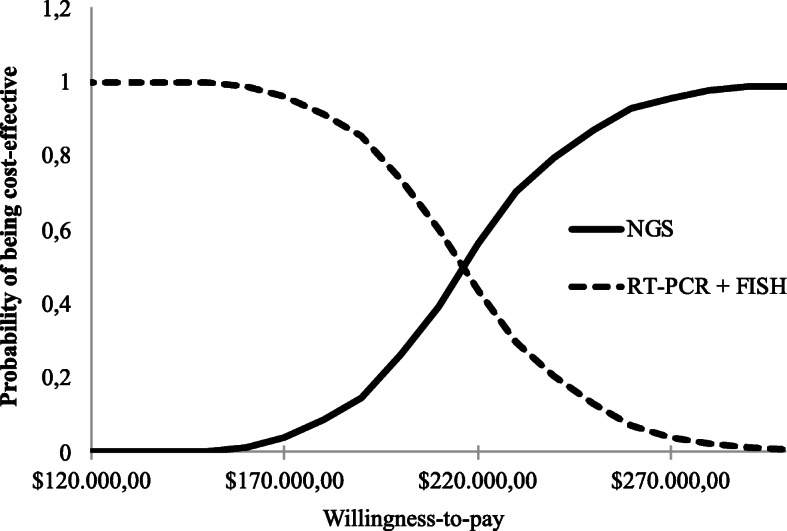
The cost-effectiveness acceptability curves showing the chance of obtaining net benefits with the NGS compared to standard strategies, at different hypothetical willingness-to-pay thresholds

Cost-effectiveness acceptability curves were constructed to show the probability of each strategy being considered as cost-effective in relation to another, using values that health plans could afford to pay per QALY (Fig. 4). Our results show that the probability of the NGS test being cost-effective is very low, less than 40% for amounts that exceed willingness to pay for reasonable QALYs in several countries (Fig. 4).

## Discussion

This study represents the first economic evaluation analyzing NGS and the cost-effectiveness ratio from the perspective of the private health insurance sector in Brazil. Economic assessments focusing on the comparison of NGS use with other tests for gene changes present in lung cancer are still relatively scarce in the literature [31] with most cost-effectiveness studies focusing on isolated biomarkers [34, 42–46]. The tests available, and the test sequence employed, may differ in their ability to accurately select patients who will likely benefit from target therapy. Therefore, our analysis sought to identify the best test-treatment strategy, from the options available in Brazil, regarding cost-effectiveness.

One of the outcome measures defined for this study considered the number of cases correctly identified by each of the diagnostic strategies. The results showed that the single test with NGS had a greater likelihood of producing a correct diagnosis (summation of true positive and negative cases) — 96.3% — than the diagnostic routine based on individual tests for EGFR, ALK and ROS1 (68%) and the test for EGFR, followed by simultaneous testing for ALK and ROS1 (72.6%). To this end, the high sensitivity, and specificity of the NGS, in both cases exceeding 99%, outperformed sensitivity compared to the EGFR test used (97.5%), however with less specificity (86.7%). The sensitivity and specificity of the FISH test for ALK and ROS1 did not compete for differences in the number of cases correctly diagnosed, since they were considered in the case of reference as being 100%. In this sense, although the number of false-negative patients for EGFR is small (depending on the sensitivity of the test) the smaller specificity leads to a greater number of false-positive results for EGFR, which, in practice, results in the loss of detection of possible cases with ALK or ROS1 mutations.

Some reports indicated that NGS surpasses Sanger sequencing in terms of sensitivity. For example, using 80 small routine samples from routine biopsies and cytology, De Biase compared NGS with Sanger sequencing, and demonstrated that NGS improved detection of EGFR mutations, particularly in samples with low tumor cell content [47]. In the present study, the ability of NGS to correctly diagnose 24% more cases of gene changes compared with other modelling strategies was associated with a great cost per individual (an average of US$1874.37 versus US$1053.49 for strategies 2 and 1, respectively). These cost differences (of approximately US $800.00 or more, which is ≥77%) response for the incremental cost-effectiveness ratio (ICER) for NGS versus strategy 2 of US$ 3479.11 per correctly diagnosed case detected.

The cost-effectiveness study modelled the insertion of the recommended therapies and estimated long-term consequences of the different test-treatment strategies. The objective was to establish a link between the diagnostic performance, its clinical value (regarding the impact on progression-free survival time and overall survival), and the incremental cost-effectiveness ratio. Thus, when the treatments and their results were incorporated into the model, the effectiveness of the tests were diluted between arms. The NGS was more effective than the other two diagnostic strategies, with small gains in years of life and quality adjusted life years (incremental gain of 0.015 in life years and 0.009 in QALYs). It was also more costly (incremental cost of US $ 1930.00), resulting in a high ICER, about US $ 214,000.00 per QALY gained. The small gain in terms of years of life and QALYs can be explained by small differences in the overall survival between treatments. Although most TKI-related clinical trials show statistically significant differences in tumor response rate (ORR) and progression free survival (PFS), they are not able to demonstrate significant differences in overall survival (OS) between these and standard chemotherapy [48].

The lack of association between the differences observed in the ORR and PFS, and the result in terms of OS is attributed, among others, to crossover between arms of clinical trials, with migration of patients from the control groups to the intervention arm, in the presence of certain predefined events such as disease progression. If survival is extended to patients who migrate between the randomized arms outcomes, such as OS, the differences could be reduced in such a way that there is no longer any statistical significance. The intention-to-treat analyses may underestimate the advantages in terms of OS and the economic efficiency of the new intervention [39].

The differences in testing costs are much more significant and impactful, and even the lower limit of the price range for the NGS practiced in the Brazilian market (US$ 1502.51) far exceeds the upper limits of the bands raised for the other two tests (respectively, US$ 477.39 and US$ 753.77). New technologies tend to be introduced in the market at higher values, with their prices tending to decline as they become more widespread. This may also occur with the NGS, with future price reductions below the lower limit currently used, which could make the strategy based on this test more favourable.

Another factor that increases the advantage of NGS over other tests is the ability to generate more information in a single test [49]. A Brazilian cohort study showed that NGS was able to identify 12% more individuals with genetic alterations that already have target therapies available in clinical practice, increasing the chances that more patients will benefit from them [40]. NGS validation has also been increasing for use in other types of biopsies, such as liquid biopsy, allowing for the identification of several genetic alterations from circulating DNA in plasma.

Another important aspect regarding treatment costs refers to the *bias* resulting from the patients living longer and adding costs to the model in relation to treatment, since in the first line with target therapy patients use the drug daily until progression. Thus, the individuals who contribute mostly to the strategy, in terms of survival, also contribute for the higher costs. Importantly, the construction of cost-effectiveness acceptability curves allows for summarization of the uncertainties about the estimates made. In this case, the acceptability curve makes it clear that the probability of the NGS test being more cost-effective than the other diagnostic-treatment strategies is quite small: less than 40% for values that exceed US$ 214,000.00 per QALY.

Doble et al. 2017, focusing on the fourth-line treatment of metastatic lung adenocarcinoma, compared three diagnostic-treatment strategies: (i) use of NGS and treatment with target therapies only in patients with detected changes, with the remaining receiving chemotherapy (vinorelbine) or supportive care; (ii) no additional tests, with chemotherapy, and (iii) no additional test with supportive treatment [35]. A decision tree combined with a Markov model was used to compare costs, years of life and QALY over a ten-year time horizon, from the perspective of an Australian health care funder and they concluded that NGS was not cost-effective. In addition, sensitivity analyses did not show trend changes in these results, which in all cases persisted above an AU$ 200,000.00/ QALY. Only the reduction in target therapy costs (in this case, used off-label because was not covered by the 4th line payment system) indicated an ICER that was more NGS favourable, but still high (220,807.00 Australian dollars/ QALY).

Some important aspects related to the tests were not incorporated into our model. An important assumption underlying this model is that the tests lead to different treatments, ignoring other factors that could contribute to variations in the outcome of therapy such as the time for the availability of test results, relevant to initiating therapy. Some international guidelines recommend EGFR and ALK results to be available within a maximum of 2 weeks (10 working days), from receipt of the specimen in laboratory [2, 3]_._ This waiting time was not included in the present study. It should be noted that although NGS is a complex test [49], its clinical usefulness in patients with lung cancer showed that the mean time between receipt of the sample at the laboratory and the results release was 7 days, and that in 78.4% of the cases, the result of the NGS was released within 10-business days [41]. However, this time was higher in the study of Hagemann in 2015 (median time of 21 days), which may be attributed to the platform used (Illumina vs Ion Torrent) [50]. In addition, another limitation is the fact that the model presented evaluated the testing specific to EGFR, ALK, and ROS1, since those are the most common actionable alterations and the only ones that had matched therapies available in Brazil.

For all the diagnostic strategies examined, the probability of not obtaining the result was considered, either due to insufficient tumour cells or due to poor sample quality. The simulations considering 1000 tests showed that NGS would result in 34 inconclusive tests, a number well below strategy 1, where testing would result in 130 inconclusive cases for EGFR mutation and 55 cases with FISH. The better performance of the NGS over other tests could be explained by its ability to detect changes in lower sample concentrations. Le Mercier (2015) [41] showed that only 1 out of 234 samples could not be tested due to insufficient material for analysis and that only 10 out of 233 samples tested were not informative, giving a success rate of 96%. Hageman’s (2015) study also showed a high success rate (97%) in tissue samples tested for lung cancer [50]. For the Brazilian reality, we consulted a laboratory that presented a cohort of 298 applications for the NGS, contemplating a rate of 95% test success [40].

In the present study, in all cases of unavailable outcome, the individual followed directly to treatment with conventional chemotherapy for the first line, not being defined a second tumour biopsy and molecular retesting since this was considered as not being the most frequent pattern of care in Brazil and also because the material obtained in the repeated biopsy could also be insufficient or inconclusive. Repeated biopsy and retesting could result in additional costs for the three screening strategies. Given the large cost-per-test differences, it is unlikely that this would change the ICER to levels that could be considered acceptable. An internationally agreed standard for sequencing analysis and data interpretation for clinical, public health, and regulatory purposes must be developed to make NGS more reliable [51]. The use of utility measures from the international literature is another limitation to be pointed out. Utilities relative to each health condition, and presence or absence of serious adverse events were obtained from cost-effectiveness studies of patients with NSCLC under TKI treatment and chemotherapy. The lack of Brazilian studies concerning impacts on the quality of life associated with different treatments justified this use and reinforces the importance of further work focusing on these measures.

The effort to use prevalence values for the various genetic alterations from national studies, and the choice to make a microsimulation model to design the gains in survival between the different strategies, are important strengths of our analysis. Although the number of studies in our country is quite small, the significant regional differences indicated justify efforts to search for and use measurements based on the Brazilian population. Likewise, the attempt to obtain data closer to that practiced in the market for both comparative tests and from a set of commercial companion tests. We also sought to aggregate drug costs used in pre-chemotherapy and room rates according to the infusion time of each chemotherapy protocol. The microsimulation model was a good choice to simulate a cohort of individuals, from their respective attributes, according to the survival curves present in the literature. In other models, such as Markov transition state models, it is possible to aggregate survival data, nevertheless assumptions must be made such as grouping individuals in the same health state. Thus, the costs and outcomes are calculated from each state, which may result in losing information at the individual level.

## Conclusions

The NGS has been finding barriers to its coverage not only in precision therapy, but also in screening for hereditary cancer, among other indications. Although the benefits of a larger diagnosis are recognized, this diagnostic model contrasts with the usual single test/single result payment model in health systems. For the payer, generating information beyond what is needed immediately in the assistance may be a source of uncertainties as the economic impact that these changes may produce.

The data presented indicated that, although the molecular diagnosis by NGS of patients with NSCLC with advanced stage adenocarcinoma histology allows for a greater number of correctly diagnosed gene alterations cases, this test was not cost-effective in terms of quality-adjusted life year from the perspective of the Brazilian supplementary health system. NGS was not deemed cost-effective compared with individual directed genomic tests for EGFR, ALK, and ROS1 in a model reflective of the current therapeutic limitations available in Brazil. As new therapies for these genomic alterations and others become available, the value of NGS testing should be reconsidered. It is possible that in the near future, the NGS may aggregate more diagnostic information that positively impacts treatments’ effectiveness in addition to optimizing the use of other types of biopsy. Considering this increase in effectiveness, and the possibility of reducing the cost of testing through its expanded use, in a short time the results of the NGS can become cost-effective or even cost-saving.

This study is part of an effort to integrate discussions related to the companion tests for detection of gene alterations and target therapy in the Brazilian health system. Nevertheless, other studies should be performed integrating liquid biopsies and a panel of comprehensive mutations related to rarer genetic alterations that possess useful target drugs.

## Data Availability

The datasets used and/or analysed during the current study are available from the corresponding author on reasonable request.

## Abbreviations

NSCLC: Non -small cell Lung Cancer
NGS: Next Generation Sequencing
ICER: Incremental cost-effectiveness ratio
OS: Overall survival
ORR: Overall response rate
TKI: Tyrosine Kinase Inhibitors
IHC: Immunohistochemistry
PFS: Progression free survival
FISH: In situ hybridization
